# Gold Nanoparticles Coated With the Antimicrobial Peptide Os‐C(W5): Anticandidal and Biological Activity

**DOI:** 10.1002/psc.70117

**Published:** 2026-08-02

**Authors:** P. J. Palm, R. R. Chirombo, C. K. Chiramba, J. C. Serem, H. Taute, M. van der Walt, A. R. M. Gaspar, M. J. Bester

**Affiliations:** ^1^ Department of Anatomy, Faculty of Health Sciences University of Pretoria South Africa; ^2^ Department of Biochemistry, Genetics and Microbiology, Faculty of Natural and Agricultural Sciences University of Pretoria South Africa

**Keywords:** antifungal, antimicrobial, gold nanoparticles, peptides

## Abstract

*Candida albicans* is a critical‐priority fungal pathogen due to its global burden of infection, biofilm‐forming ability, and increasing drug resistance. Previously, the tick‐derived antimicrobial peptide (AMP), Os‐C, demonstrated antifungal, antioxidant, and nitric oxide (NO) scavenging activity. Subsequent tryptophan end‐tagging produced Os‐C(W_5_) which improved antifungal activity and reduced salt sensitivity; however, the proteolytic susceptibility of Os‐C(W_5_) remained a limitation.

Researchers have shown that N‐terminal tryptophan tagging of a gold binding peptide generated gold nanoparticle (GNPs) of a clinically relevant size. We successfully applied this method with minor modifications to generate GNPs coated with Os‐C(W_5_). GNP@Os‐C(W_5_) exhibited an average diameter of 14.2 ± 0.32 nm, with preserved peptide disorder in peptide secondary structure after conjugation. Compared with free Os‐C(W_5_), GNP@Os‐C(W_5_) activity against planktonic *C. albicans* was reduced, but antibiofilm activity was maintained, associated with a reduction in biofilm biomass. Ultrastructural changes to planktonic *C. albicans* included roughened and irregular surfaces, membrane indentations, and extracellular debris indicating cell lysis. Samples treated with Os‐C(W5) and GNP@Os‐C(W5) showed visibly less dense biofilm architecture and fewer apparent hyphal structures relative to the untreated control. Both Os‐C(W_5_) and GNP@Os‐C(W_5_) were non‐cytotoxic to HaCat cells. Most importantly, resistance to the protease trypsin was increased. However, antioxidant properties of Os‐C(W_5_) were lost with GNP conjugation, whereas both Os‐C(W_5_) and GNP@Os‐C(W_5_) compared with Os‐C lacked NO scavenging activity. Overall, this method of GNP conjugation provides a viable strategy to achieve enhanced AMP stability, retained antifungal activity, although the ability of GNP@Os‐C(W_5_) to reduce oxidative stress was compromised.

## Graphical abstract

Gold nanoparticles conjugated with a tick‐derived antimicrobial peptide, Os‐C(W5), demonstrate enhanced proteolytic stability and potent antifungal activity against *Candida albicans* biofilms and planktonic cells. These non‐cytotoxic nanoconjugates represent a promising biocompatible strategy for combating resistant fungal infections while maintaining high stability in biological environments.

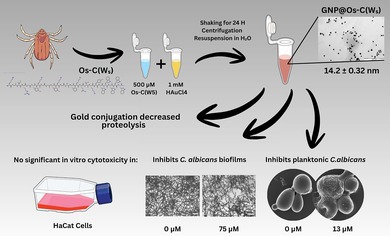

## 1 Introduction

Fungi are common residents of the human microbiome and are found on all our mucosal surfaces [1]. In immunocompromised individuals, this otherwise balanced relationship can break down, leading to fungal overgrowth and opportunistic infections that may be superficial, or systemic [2]. Currently, the treatment of fungal infections relies on only three major classes of antifungal drugs: polyenes, azoles, and echinocandins. Each class is associated with notable adverse effects and increasing reports of antifungal resistance (AFR) [3]. Unlike many other infectious diseases, this narrow therapeutic arsenal offers few alternatives when resistance emerges, greatly limiting treatment options. The global rise in AFR represents a particularly serious clinical challenge, highlighting the need for antimicrobial stewardship alongside the development of new antifungal drugs and innovative treatment strategies to effectively combat AFR [4].

Various antimicrobial peptides (AMPs) exhibit antifungal activity, and as of September 2025, the Antimicrobial Peptide Database (APD6) contains 1812 antifungal peptides (AFPs) of which 1093 have anticandidal activity [5]. Generally, AFPs target the fungal cell membrane; however, additional cell wall components, including chitin, β‐glucan, and mannan, have also been identified as targets [6]. Beyond membrane and cell wall disruption, AFPs can act on intracellular pathways, inducing reactive oxygen species (ROS) production [7], mitochondrial dysfunction, and cell cycle disruption [8]. Furthermore, combinations of AFPs with conventional antifungals can produce synergistic effects. Notably, BLfcin and Lf [1, 2, 3, 4, 5, 6, 7, 8, 9, 10, 11] enhanced the efficacy of amphotericin B against resistant *C. albicans* strains [6].

Despite therapeutic promise, the clinical translation of AMPs is frequently hindered by susceptibility to high salt concentration, serum components, and proteolytic degradation [9]. One strategy to overcome these challenges involves end‐tagging AMPs with tryptophan residues, which enhances hydrophobic interactions with fungal membranes to improve antifungal potency [10]. This strategy was successfully applied to the tick‐derived AMP, Os‐C, where the tryptophan end‐tagged Os‐C(W_5_) exhibited antifungal activity in a physiologically salt environment. However, susceptibility to proteases was still a challenge [7].

An alternative strategy to improve peptide stability is to attach AMPs to gold nanoparticles (GNPs), which limits protease access to cleavage sites within the peptide sequence [11]. This approach offers the advantage of preserving the native peptide sequence while exploiting the high stability and biocompatibility [12]. In addition, GNPs can act as effective drug delivery vehicles, allowing for increased interaction with microbial membranes and controlled release of conjugated antimicrobial agents in response to exposure of internal or external stimuli such as pH changes, glutathione levels, and light exposure [13, 14, 15].

GNPs functionalized with chemical linker molecules that act as capping agents have been widely employed to attach peptides onto the GNP surface. In a study by Casciaro et al. (2017), Esculentin‐1a(1–21)NH_2_ was covalently conjugated to ~14 nm PEG‐linked GNPs, resulting in a 15‐fold increase in activity against *Pseudomonas aeruginosa* without mammalian cytotoxicity, along with enhanced protease resistance and salt tolerance [16]. Similarly, Palmieri et al. (2018) reported a four‐fold increase in antibacterial and antibiofilm activty for the synthetic peptide 1018 K6 conjugated to PEG‐stabilized GNPs agains *Listeria monocytogenes* and *Salmonella typhmurium* [17]. In fungi, Rahimi et al. (2019) demonstrated that indolicidin covalently attached to GNPs reduced the MIC_50_ against planktonic *C.albicans* from 26.23 μM to 6.65 μM across 10 clinical isolates [18].

Peptides can also attach non‐covalently to GNPs, where specific amino acids act as reductants to convert Au^3+^ to Au^0^, initiating nanoparticle nucleation and growth. Furthermore, non‐covalent interactions between the peptide and growing GNPs control particle size in a similar manner to natural biomineralization [19]. Hydrophobic aromatic residues, such as phenylalanine, tyrosine, and tryptophan, efficiently reduce Au^3+^ and adsorb onto gold surfaces by positioning their aromatic rings parallel to the GNP surface [20, 21], whereas positively charged lysine and arginine residues stabilize nanoparticles and promote aqueous dispersibility through Au‐N interactions [22].

Ozaki et al. (2020) demonstrated the successful formation of GNPs coated with the 11 amino acid, gold‐binding peptide AuBP1, where increasing the number of N‐terminal tryptophan residues resulted in the formation of progressively smaller nanoparticles, with average diameters of 22.8 ± 6.3, 11.7 ± 2.2 and 8.6 ± 1.8 nm, respectively [23]. These sizes fall within the range considered suitable for clinical application. Inspired by this, we propose that Os‐C(W_5_) can similarly mediate the formation of GNPs as a reducing and capping agent via terminal tryptophan‐driven reduction of Au^3+^ [24], followed by stabilization via lysine‐ and arginine‐residues [25], generating antifungal peptide‐coated nanoparticles. Due to the non‐covalent nature of these peptide interactions (Table 1), GNP@Os‐C(W_5_) may serve as a delivery platform, facilitating peptide interaction with the fungal cell wall. Accordingly, the aim of this study was to synthesize and characterize the structural features and bioactivity of GNP@Os‐C(W_5_) relative to Os‐C(W_5_).

**TABLE 1:** Physicochemical properties of peptides used in the study.

Peptide	Sequence	Molecular weight^a^ (g/mol)	Charge^a^	pI^a^	Hydrophobicity^b^ (%)
Melittin	GIGAVLKVLTTGLPALISWIKRKRQQ‐NH_2_	2846.48	+5	12.6	50
Os	KGIRGYKGGYCKGAFKQTCKCY	2459.92	+6	10.0	13.6
Os‐C	KGIRGYKGGY_KGAFKQT_K_Y^c^	2150.49	+6	10.8	15.8
Os‐C(W_5_)	KGIRGYKGGY_KGAFKQT_K_Y**WWWWW** ^c^	3081.55	+6	10.8	33.3

^a^ Calculated using GenScript peptide molecular weight calculator. [https://www.genscript.com/tools/peptide‐molecular‐weight‐calculator].

^b^ Calculated using peptide 2.0. [https://www.peptide2.com/N_peptide_hydrophobicity_hydrophilicity.php].

^c^ Underscores represent missing cysteine residues.

## 2 Material and methods

### 2.1 Peptides

Melittin, Os, Os‐C, Os‐C(W_5_) were obtained from GenScript (Piscataway, New Jersey, USA) and synthesized using FlexiPeptide technology. The peptides were analyzed for purity and molecular masses by the vendor using reverse‐phase high performance liquid chromatography and mass spectrometry, respectively (Figure S1–S4). Sequences and physicochemical properties are shown in Table 1.

Lyophilized peptides were resuspended in a volume of 200 μL double distilled water (ddH_2_O), and the absorbance was measured at 280 nm (UV–Vis SP300, Optima, Tokyo, Japan). The concentration of the peptides was calculated using the following equation:

$$c=\frac{\mathrm{MW}\times \mathrm{df}\times \mathrm{Abs}}{\mathrm{no}.\mathrm{Tyr}\left(\mathrm{Ex}\right)\times \mathrm{no}.\mathrm{Trp}\left(\mathrm{Ex}\right)}$$

where ***c*** is the peptide concentration in mg/mL, MW is the peptide molecular weight in g/mol, df is the dilution factor and Abs is the absorbance at 280 nm. The extinction coefficient of tyrosine (*Tyr*) is 1200 AU/mmol/mL and tryptophan (*Trp*) is 5560 AU/mmol/mL [26]. Stock solutions were prepared in sterile, filtered ddH_2_O and stored at −20°C.

### 2.2 Synthesis and Characterization of AuNP

GNPs were synthesized according to the methods of Ozaki et al. (2020) with slight modifications [23]. In a microcentrifuge tube, 210 μL ddH_2_O was added to 105 μL of 500 μM Os‐C(W_5_). To this mixture, 35 μL of 1 mM HAuCl_3_ **•**2H_2_O was added to the Os‐C(W_5_) solution to achieve a final concentration of 150 μM. The tube was wrapped in aluminum foil and placed on a Pelco R2 rotary mixer (Pelco; Fresno, California, USA) at a speed of 50 rpm overnight. A UV–Vis spectrum scan between 200–600 nm was then undertaken to identify the surface plasmon resonance (SPR) peak associated with GNPs of 10–40 nm [27], and to calculate the GNP concentration at the maximum of 535 nm using the Lambert–Beer law:

$$\text{Absorbance}\left(535\mathrm{nm}\right)=\varepsilon \times p\times M$$

where *ε* is the molar coefficient of GNPs with an average diameter of 14 nm (1.76 × ^108^M − ^1^.cm^−1^) at 535 nm [28], *p* is the pathlength in cm and *M* is the molar concentration in mol/L.

To determine the concentration of the bound GNP@Os‐C(W_5_), the mixture was centrifuged at 18,000 ×*g* for 30 min. The supernatant was collected, and the concentration of unbound Os‐C(W_5_) was determined as described above. The total GNP bound peptide concentration was calculated as follows:

$$\begin{array}{c} \left[\mathrm{Os}-C\left(W_{5}\right)\right]\;\text{bound to}\;\mathrm{GNP}=\left[\mathrm{Os}-C\left(W_{5}\right)\right]\;\text{reaction}\\ -\left[\mathrm{Os}-C\left(W_{5}\right)\right]\text{supernatant} \end{array}$$

The concentration of the bound Os‐C(W_5_) was used for all subsequent anticandidal, cytotoxicity, and anti‐inflammatory studies.

As a negative control, GNP‐bound tyrosine (GNP@Tyr) was prepared using the method described by Si and Mandal (2007) with modifications [24]. To 10 mM L‐tyrosine in 475 μL ddH_2_O, dropwise, 25 μL of 100 mM HAuCl_3_·2H_2_O was added. The mixture was vortexed for 2 min before adjusting the pH to 11 using sodium hydroxide. Afterward, the tube was wrapped in aluminum foil and placed on a Pelco R2 rotary mixer (Pelco, California, USA) at a speed of 50 rpm overnight. The formed GNPs were collected by centrifugation at 18,000 ×*g* for 30 min and were then resuspended with 350 μL ddH_2_O. A UV–Vis spectrum scan between 200–600 nm was used to identify the SPR peak and to calculate the GNP concentration using the Lambert–Beer law as described above for GNPs of 14 nm.

For all experiments, the GNP@Tyr concentration used represents the GNP content/concentration of GNP@Os‐C(W_5_). Transmission electron microscopy (TEM) was used to determine the particle sizes of GNP@Os‐C(W_5_) and GNP@Tyr. According to the method described by Ozaki et al. (2020) 20 μL of each GNP preparation was placed on a copper TEM grid covered with a Nisshin EM colloid membrane (Nisshin, Tokyo, Japan) for 10 min. Filter paper was used to soak up excess solvent. Grids were gently washed three times using ddH_2_O, blotted dry, and dried overnight in a desiccator. Samples were imaged on a JEM‐2100F TEM (JEOL, Tokyo, Japan) at 115 kV. Particle diameters (*n* = 100 per experiment) were measured using ImageJ v 1.6 (National Institutes of Health, USA).

### 2.3 Circular Dichroism Spectroscopy

Circular dichroism (CD) spectroscopy was used to determine the secondary structure of Os‐C(W_5_) relative to GNP@Os‐C(W_5_). In a 0.2 cm path length quartz cuvette, 50 μM Os‐C(W_5_) and GNP@Os‐C(W_5_) were dissolved in either 5 mM Tris buffer or 50 mM sodium dodecyl sulfate (SDS). A spectrum was generated using a J‐1500 CD‐spectrophotometer (Jasco; Easton, Maryland, USA) and scans were performed at 20°C, over the 180–260 nm range, with a path length of 0.2 cm, a scan speed of 100 nm/min, data pitch of 0.5 nm, and a bandwidth of 2 nm. The mean residue ellipticity of the peptides was calculated using the following equation:

$$\left[\theta \right]=\frac{\theta }{\left(10\;x\;c\;x\;l\;x\;n\right)}$$

where [*θ*] is the mean residue ellipticity in deg.·cm^2^·dmol^−1^, θ is the ellipticity in mdeg, *l* is the path length in cm, *c* is the peptide concentration in mol/L, and *n* is the number of residues in the peptide.

### 2.4 Antifungal Activity

To determine the 50% minimum inhibitory concentration (MIC_50_) of Os‐C(W_5_) and GNP@Os‐C(W_5_) against *C. albicans*, a modified version of the European Committee on Antimicrobial Susceptibility Testing (EUCAST) method [29] was employed. *Candida albicans* (ATCC 90028) cells were grown to exponential phase, washed twice with RPMI‐1640, and adjusted to 1 × 10^5^ CFU/mL. Fifty microliters of the cell suspension was added to polypropylene 96‐well plates (Greiner Bio‐One, Kremsmunster, Austria) and treated with serial dilutions of miconazole (0.313 μM—80 μM; positive control), Os‐C(W_5_) (0.625 μM—40 μM), and GNP@Os‐C(W5) (0.267 μM—68.4 μM). Cell viability was determined with the resazurin assay [30]. After 2 h of incubation, 10 μL resazurin was added (made up in 0.1 M phosphate buffered saline), followed by a further 1‐h incubation at 37°C. Fluorescence was then measured at Ex/Em 535/590 nm using a Spectramax Multi‐Mode Microplate Reader (Molecular Devices; San Jose, California, USA). Percentage inhibition was calculated and MIC_50_ values were obtained from sigmoidal dose–response curves.

To determine the minimum fungicidal concentration (MFC), the colony forming unit (CFU) assay was performed according to the method described by Nordin et al. (2012) [31]. After 3 h of incubation as described above, 100 μL of each suspension was directly plated onto yeast, peptone and dextrose (YPD) agar plates. The growth control was diluted 1 000 times in 10 mM sodium phosphate (NaP) buffer (pH 7.4) before plating. Plates were incubated for 48 h at 30°C and the number of visible colonies in each plate was counted.

The 50% biofilm inhibition concentration (BIC_50_) was determined as described by Chiramba et al. (2024) [7]. Briefly, several single colonies grown on YPD agar were collected and suspended in YPD broth (0.5% (w/v) yeast extract, 1% (w/v) peptone, 2% (w/v) dextrose) and incubated for 18 h at 30°C. The cells were collected by centrifugation, washed, and then resuspended and diluted in RPMI‐1640 to a cell density of 2 × 10^6^ CFU/mL. Cell aliquots of 100 μL were added to the wells of a flat‐bottom 96‐well polystyrene plate. Increasing concentrations of 100 μL miconazole (0.97 μM—125 μM; made up in 0.5% dimethyl sulfoxide (DMSO)‐RPMI‐1640), GNP@Tyr (0.39 mM—2.5 mM; equivalent to the GNP concentration in GNP@Os‐C(W_5_)) and GNP@Os‐C(W_5_) (0.5 μM—75 μM; equivalent to the Os‐C(W_5_) concentration in GNP@Os‐C(W_5_)) in ddH_2_O were added to the wells. After 24 h incubation at 37°C, followed by a wash step with 0.1 M PBS, the biofilm viability and biomass was determined with the resazurin (as described above) and crystal violet (CV) assays respectively.

For the CV assay, the biofilms were washed with phosphate buffered saline (PBS) and fixed with 20% (v/v) formaldehyde at room temperature for 20 min. The fixative was removed, and the plates were dried thoroughly before staining with 0.1% (m/v) CV (Sigma‐Aldrich; Johannesburg, SA) for 20 min. The excess dye was removed, and the wells were then rinsed with ddH_2_O and dried before images were taken with an inverted light microscope equipped with a camera (Optika Microscopes; Ponteranica, Italy). The CV dye was then extracted with 30% (v/v) acetic acid for 15 min at room temperature before the absorbance of the extracted dye was determined at 550 nm.

### 2.5 Scanning Electron Microscopy

Scanning electron microscopy (SEM) was employed to determine the effects of Os‐C(W_5_) and GNP@Os‐C(W_5_) at the MIC_50_ on the morphology of *C. albicans* cells. For SEM, the cells were attached to glass coverslips coated with poly‐L‐lysine. The round 10 mm diameter coverslips (Lasec; Johannesburg, SA) were washed in 10% NaOH solution (w/v) and 20% ethanol (v/v) with shaking for 2 h, rinsed several times in ddH_2_O, sterilized with 99.9% ethanol (v/v) for 30 min and then air dried. The coverslips were then immersed in poly‐L‐lysine (7000–15,000 kDa, Sigma‐Aldrich; Johannesburg, SA) for 2 h, rinsed again in ddH_2_O, and dried for at least 3 days under sterile conditions.

After exposure to 1.37 μM Os‐C(W_5_) and 13.21 μM GNP@Os‐C(W_5_) for 3 h, 50 μL of 2.5% glutaraldehyde/formaldehyde fixative was added to the cells for 20 min and then 100 μL of the cell suspension was placed on the poly‐L‐lysine coated coverslips for 70 min. The coverslips were then rinsed with 0.075 mM NaP buffer (pH 7.4) to remove any unattached cells. A volume of 300 μL of 1% osmium tetroxide was then used to post‐fix the cells for 45 min, after which the coverslips were rinsed with 0.075 mM NaP buffer. Samples were dehydrated with 30%, 50%, 70% and 100% ethanol followed by drying with two drops of hexamethyldisilane (HMDS) overnight [32]. The fixed samples were mounted with carbon tape onto aluminum stubs coated with carbon and viewed at high resolution with an Ultra Plus FEG SEM (Zeiss; Oberkochen, Germany).

### 2.6 Cytotoxicity

Cytotoxicity was evaluated using the HaCaT cell line, a human immortalized non‐tumorigenic keratinocyte cell line [33]. A volume of 90 μL, HaCaT cells, 1 × 10^5^ cells/mL, were plated in a 96‐well plate and then cultured in Dulbecco's Modified Eagle's Medium (DMEM), supplemented with 2 mM L‐glutamine, 10% (v/v) fetal bovine serum (FBS), 2 mM L‐glutamine and 1% (v/v) penicillin/streptomycin/fungizone (DMEM/FCS) for 24 h at 37°C with 5% CO_2_. The cytotoxicity of 31–500 μM Tyr@GNP and 0.35–15 μM Os‐C(W5)@GNP was determined and then the cytotoxicity of Os‐C(W5)@GNP was compared with melittin, miconazole and Os‐C(W_5_) at 15 μM. All exposures were for 24 h before cell viability was determined with the 3‐[4,5‐dimethylthiazol‐2‐yl]‐2,5‐diphenyl tetrazolium bromide (MTT) assay. To each well, 11 μL of MTT (final concentration, 1 μg/mL) solution was added and after 3 h at 37°C and 5% CO_2_, the medium was removed and the plate was dried at room temperature. The formazan crystals were dissolved in 50 μL of a 25% DMSO solution in ethanol, followed by the measurement of absorbance at 570 nm on a Spectramax Multi‐Mode Microplate Reader. The percentage relative cell viability was calculated relative to control, with no drug or peptide added.

### 2.7 Trypsin Stability Assay

Trypsin stability was assessed using the method described by Qui et al. (2017), with some minor modifications [34]. Briefly, 80 μL of melittin (0.625 μM—80 μM; positive control), Os‐C(W_5_) (0.313 μM—40 μM), and GNP@Os‐C(W_5_) (0.136 μM—40 μM) was incubated with 20 μL trypsin in PBS (37 mM NaCl, 2.7 mM KCl, 10 mM Na_2_HPO_4_, 1.8 mM KH_2_PO_4_, pH 7.4), yielding a final volume of 100 μL (final trypsin concentration = 1.86 U). Control samples included i) peptides at the same concentration incubated with 20 μL PBS instead of trypsin, and ii) 80 μL PBS with 20 μL trypsin in PBS. After 1 h incubation at 37°C, trypsin activity was terminated by heat denaturation at 60°C for 20 min. Antiplanktonic activity was subsequently determined as described above.

### 2.8 Antioxidant Assays

The Trolox equivalent antioxidant capacity (TEAC) and oxygen radical absorbance capacity (ORAC) assays were used to evaluate antioxidant activity. The TEAC reagent, consisting of 3 mM potassium peroxodisulfate solution (K_2_S_2_O_3_) and 8 mM 2,2‐azinobis‐(3‐ethylbenzothiazoline‐6‐sulfonate) (ABTS) was prepared and incubated for 12 h in the dark to form a stable blue‐green solution. A 30‐times diluted working solution of TEAC reagent in 0.1 M PBS was prepared. Concentration series of 0.1–1 mM Trolox, glutathione (GSH; positive control), Os‐C(W_5_) and GNP@Os‐C(W_5_) were prepared. A volume of 10 μL for each sample was added to the wells of a 96‐well plate, followed by 290 μL TEAC working solution, and incubated for 30 min in the dark at room temperature. The absorbance was then measured at 734 nm.

A modified method of the oxygen radical absorbance capacity (ORAC) assay described by Ou et al. (2013) was used with slight modifications [35]. A concentration range of 0.1 mM—1 mM Trolox and 0.00125 mM—1 mM GSH, Os‐C(W_5_) and GNP@Os‐C(W_5_) were prepared. A volume of 10 μL for each sample, and ddH_2_O (negative control) was added to the wells followed by 40 μL of 0.11 μM AAPH and 160 μL of 0.139 nM fluorescein. The change in fluorescence was measured at an excitation of 485 nm and an emission of 520 nm at 5‐min intervals for 2 h at 37°C.

For both antioxidant assays, Trolox calibration curves were used to calculate mM Trolox equivalents (TE) for GSH, Os‐C(W_5_), and GNP@Os‐C(W_5_). The gradients of these plots were used to determine mM TE/mM peptide.

### 2.9 Nitric Oxide Scavenging Assay

The NO scavenging assay was performed as described by Magoshi et al. (2023) [36]. A 5 mM sodium nitroprusside (SNP) solution was incubated in the light for 30 min at room temperature and then 80 μL SNP was added to a 20 μL sample (25 μM GSH, melittin, Os, Os‐C, and Os‐C(W_5_) as well as 15 μM of GNP@Os‐C(W_5_)) in a 96‐well plate and incubated for 1 h at room temperature in the dark. After incubation, the NO was quantified with Griess reagent (1% (w/v) sulphanilamide and 0.1% (w/v) *N*‐(1‐naphthyl)‐ethylenediamine dihydrochloride in 2.5% phosphoric acid) in a 96‐well plate, followed by incubation in the dark at room temperature for 10 min. Afterwards, the absorbance was measured at 570 nm. The nanomolar concentration of NO scavenged was determined using a NaNO_2_ standard curve, and the data was expressed as the nM NO scavenged/μM peptide.

### 2.10 Statistical Analysis

Each experiment consisted of three repeats of each concentration, and the data was presented as means ± standard error (SEM) of three repeats, unless stated otherwise. GraphPad Prism v6.01 (California, USA) was used to perform statistical analysis of the data. The data was evaluated for normality using the D'Agostino‐Pearson omnibus normality test. If the data presented a Gaussian distribution, one‐way ANOVA with multiple comparisons or *t*‐tests were done to determine if there was a significant difference between the compound exposure and controls. If the data did not pass the normality test, the Kruskal–Wallis nonparametric test was done followed by multiple comparisons. A *p*‐value of below 0.05 was taken as significant.

## 3 Results

### 3.1 Synthesis and Characterization of GNP@Os‐C(W_5_)

**FIGURE 1:**
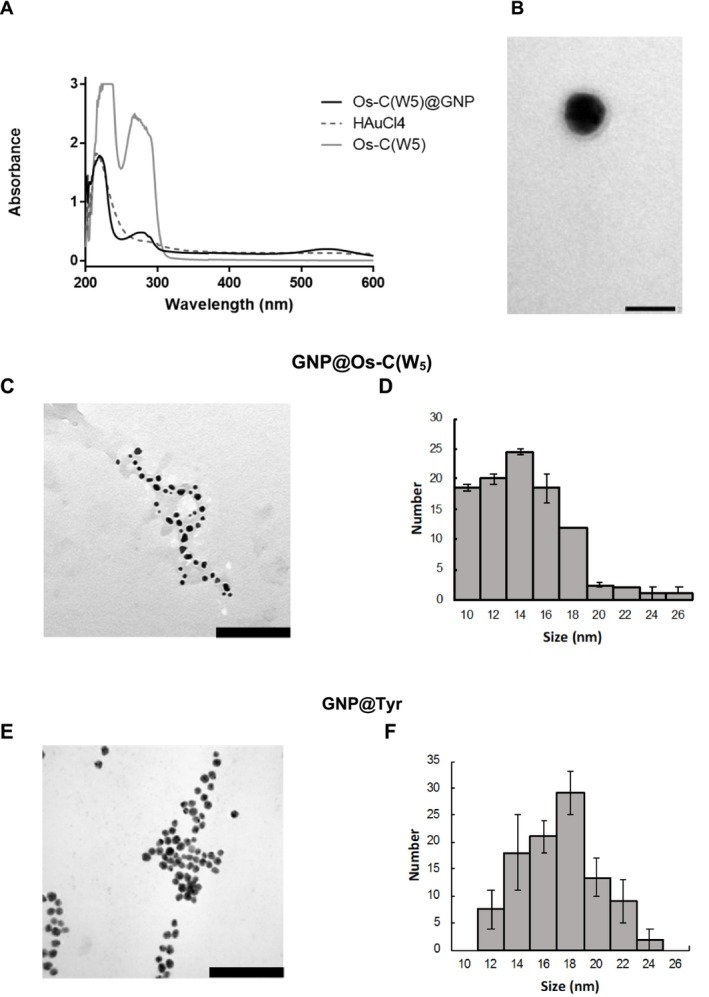
Characterization of peptide‐capped GNPs. (A) Representative UV–Vis spectra of HAuCl_4_, Os‐C(W_5_) and GNP@Os‐C(W_5_) with the SPR peak at 535 nm. (B) A high magnification TEM image of a GNP with a peptide corona. (C–D) TEM image and size distribution of GNP@Os‐C(W_5_), while (E–F) show those of GNP@Tyr. Scale bars: 20 nm for B; 200 nm for C and E.

The UV–Vis spectra of Os‐C(W_5_), HAuCl_4_, and GNP@Os‐C(W_5_) were measured between 200 nm–600 nm. For Os‐C(W_5_) (Figure 1A, gray), the peaks at 280 nm and 220 nm represent the aromatic rings of tryptophan and tyrosine and the carbonyl bonds between amino acids, respectively [37]. The HAuCl4•2H2O solution has a peak at 215 nm (Figure 1A, gray broken line). For GNP@Os‐C(W_5_) (Figure 1A, black line), the peak at 280 nm is reduced whereas the SPR peak is observed at 535 nm. For GNP@Os‐C(W_5_), the SPR maximum at 535 nm was 0.18 ± 0.02, representing 1.25 nM GNP. Peptide coupled to the GNPs is estimated to be 114.89 ± 12.01 μM, with a coupling efficiency of 76.59%. A high‐magnification TEM image reveals the presence of a corona, possibly indicating the presence of peptide, which is measured to be 2.4 ± 0.15 nm (*n* = 3) from the surface of the GNPs (Figure 1B).

To assess the importance of N‐terminal tryptophan tagging for gold reduction and stabilization, (W_5_)Os‐C alone was tested to see if this peptide could generate stable GNPs (Figure S5). An SPR peak appeared immediately after mixing, but vanished within 24 h, indicating loss of colloidal stability and nanoparticle aggregation. This rapid disappearance confirmed that (W_5_)Os‐C lacks sufficient reducing and capping capacity on its own, highlighting the essential role of the tryptophan tag in producing stable GNPs. Therefore, (W_5_)Os‐C was excluded from subsequent investigations.

TEM analysis demonstrated that GNP@Os‐C(W_5_) formed smaller, predominantly quasi‐spherical nanoparticles, whereas the GNP@Tyr control, with Tyr serving as both reducing and capping agents, displayed a more consistently spherical morphology (Figure 1). GNP@Os‐C(W_5_) exhibited an average particle diameter of 14.22 ± 0.32 nm, significantly smaller when compared with GNP@Tyr (16.20 ± 0.32 nm), indicating Os‐C(W_5_) as an efficient reducing and capping agent. The particle size distributions ranged from 10 nm—26 nm for GNP@Os‐c(W_5_) and 12 nm—24 nm for GNP@Tyr. Furthermore, increasing concentration of Os‐C(W_5_) from 37 mM to 150 mM resulted in a narrower, yet higher SPR peak, which remained lower than that observed for GNP@Tyr (Figure S6).

### 3.2 Conjugation to GNPs Induce Conformational Changes in Os‐C(W_5_)

**FIGURE 2:**
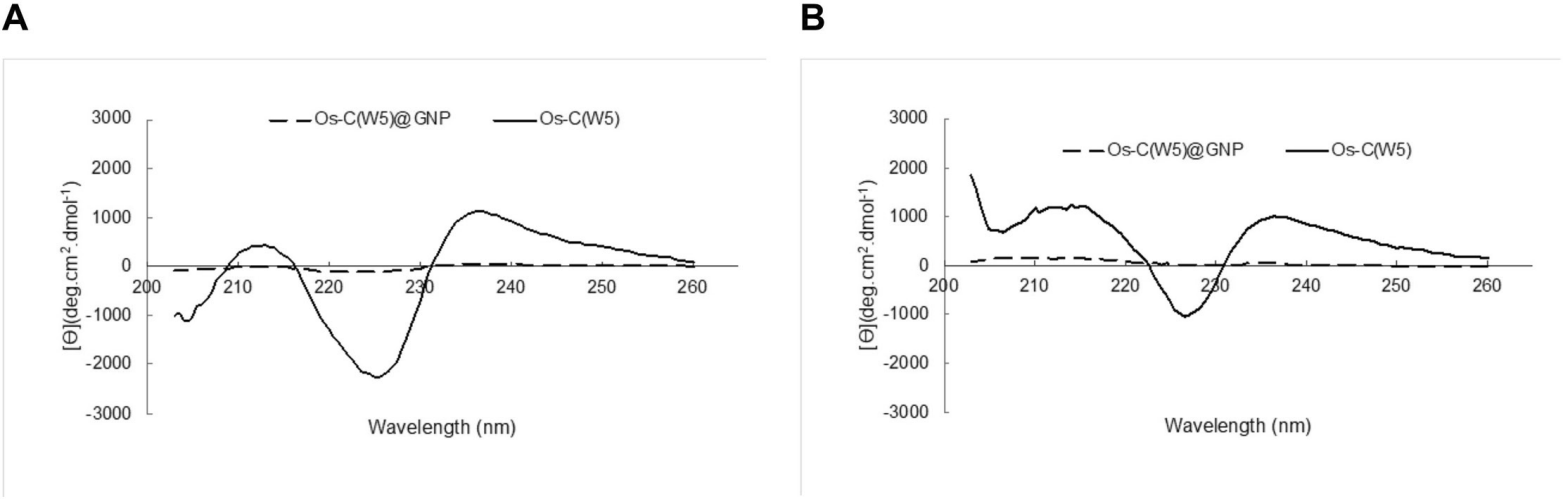
Representative UV‐CD spectra of Os‐C(W_5_) and GNP@Os‐C(W_5_) in (A) Tris buffer and (B) SDS. Spectra of 50 μM Os‐C(W_5_) and GNP@Os‐C(W_5_) in 5 mM Tris buffer and 50 mM SDS were obtained using a 0.2 cm path length quartz cuvette.

Circular dichroism spectroscopy was employed to compare the secondary structure of GNP@Os‐C(W_5_) with that of free Os‐C(W_5_) in Tris buffer and SDS, representing aqueous and membrane‐mimicking environments, respectively (Figure 2). In Tris buffer, GNP@Os‐C(W_5_) and Os‐C(W_5_) exhibited negative bands between 220 nm and 230 nm, consistent with a predominantly disordered conformation and absence of α‐helical or β‐sheet signatures [7]. However, GNP@Os‐C(W_5_) displayed a higher mean residue ellipticity value between these wavelengths. In SDS, a negative band was observed for Os‐C(W_5_) between 220 nm and 230 nm whereas GNP@Os‐C(W_5_) exhibited a mean residue ellipticity value close to zero. The absence of a pronounced negative band characteristic of Os‐C(W_5_) suggests that conjugation to GNPs causes conformational restriction with retained structural responsiveness under membrane‐mimicking conditions.

### 3.3 Conjugation to GNPs Retains Biofilm Preventing Activity of Os‐C(W_5_)

The antifungal activity of Os‐C(W_5_) and GNP@Os‐C(W_5_) against planktonic and biofilm forms of *C. albicans* was evaluated (Table 2). Os‐C(W_5_) displayed potent antiplanktonic activity, with an MIC_50_ of 1.37 ± 0.08 μM for Os‐C(W_5_), significantly lower than miconazole (7.04 ± 0.94 μM). In contrast, GNP@Os‐C(W_5_) exhibited a statistically higher MIC_50_ of 13.21 ± 0.15 μM, indicating that although activity is retained upon conjugation, it is reduced compared with free Os‐C(W_5_).

**TABLE 2:** Antifungal activity of Os‐C(W_5_) and GNP@Os‐C(W_5_) against *C. albicans* .

	Antiplanktonic activity		Biofilm prevention	
			Cell viability	Cell biomass
Compound	MIC_50_ (μM)	MFC (μM)	BIC_50_ (μM)	BIC_50_ (μM)
Miconazole	7.04 ± 0.94	20.01	2.47 ± 0.88	—
Os‐C(W_5_)^a^	1.37 ± 0.08*	10.03	11.2 ± 3.69	10.6 ± 3.77
GNP@Os‐C(W_5_)	13.21 ± 0.15*	34.02	8.93 ± 1.24	5.99 ± 2.28
GNP@Tyr	NI	NI	NI	NI

*Note:* Data shows the average findings of three independent biological repeats conducted in triplicate ± SD.

Abbreviation: NI = No Inhibition.

* Denotes significant difference between the MIC_50_ of miconazole and the other compounds (*p* < 0.05).

^a^ Value obtained from [7].

A similar trend was observed for fungicidal activity where the MFC values were 10 μM for Os‐C(W_5_), 20 μM for miconazole, and 34 μM for GNP@Os‐C(W_5_), confirming that GNP conjugation reduces potency against planktonic *C. albicans* . Accordingly, Os‐C(W_5_) is classified as fungistatic, as its MFC significantly exceeded its MIC, whereas GNP@Os‐C(W_5_) is considered fungicidal as fungal killing occurs at concentrations comparable to its MIC_90_ (Table S1).

Os‐C(W_5_) was previously reported to inhibit *C. albicans* biofilm formation with a BIC_50_ of 11.2 ± 3.69 μM (cell viability) and 10.6 ± 3.77 μM (biomass), under identical experimental conditions as used in the present study [7]. After conjugation onto GNPs, GNP@Os‐C(W_5_) exhibited BIC_50_ values of 8.93 ± 1.24 μM (cell viability) and 5.99 ± 2.28 μM (total biomass). Relative to free Os‐C(W_5_), this corresponds to a decrease in the BIC_50_ for cell viability and biomass of approximately 1.25‐fold and 1.77‐fold, respectively. Both miconazole and GNP@Os‐C(W_5_) inhibited *C. albicans* biofilm development in a dose‐dependent manner (Supplementary Figure S7). Overall, these results indicate that nanoparticle conjugation alters the antifungal profile of Os‐C(W_5_), reducing planktonic potency while maintaining antibiofilm activity. Nonetheless, activity is within the micromolar range indicating that effective *C. albicans* killing is achieved.

### 3.4 Exposure to Os‐C(W_5_) and GNP@Os‐C(W_5_) Leads to Morphological Changes

**FIGURE 3:**
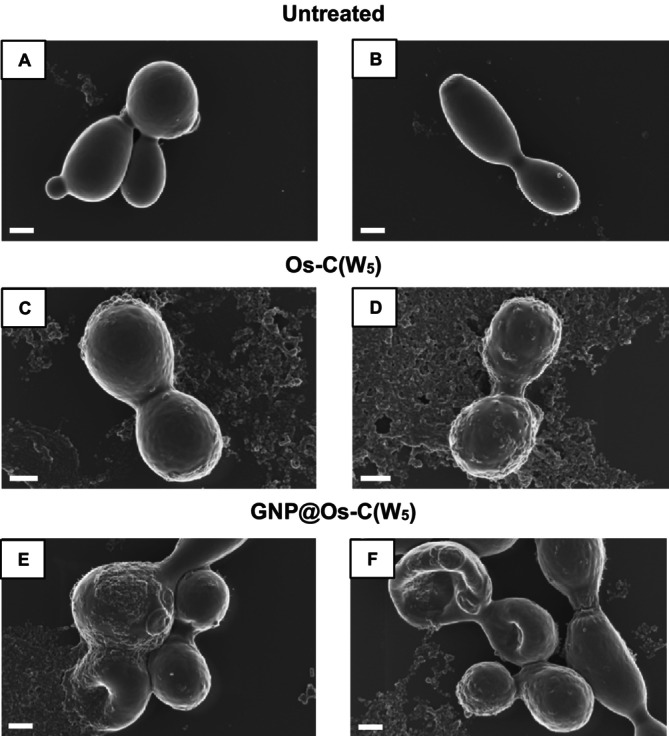
Effect of Os‐C(W_5_) and GNP@Os‐C(W_5_) on cell morphology. Planktonic *C. albicans* cells were (A–B) untreated or exposed to either (C–D) 1.37 μM of Os‐C(W_5_) or (E–F) 13.21 μM GNP@Os‐C(W_5_) for 3 h and imaged with SEM. Scale bars = 1 μm.

Scanning electron microscopy was used to compare the morphological effects of GNP@Os‐C(W_5_) and free Os‐C(W_5_) on planktonic *C. albicans* . Exponentially growing cells were exposed for 3 h at MIC_50_ concentrations of Os‐C(W_5_) (1.37 μM) and GNP@Os‐C(W_5_) (13.21 μM). The untreated cells (Figure 3A,B) displayed the typical ovoid shape with smooth, intact cell surfaces typical of *C. albicans* . In contrast, cells exposed to Os‐C(W_5_) exhibited clear signs of cell wall damage (Figure 3C,D), including roughened and irregular surfaces, membrane indentations, and extracellular debris consistent with cell lysis. Exposure to GNP@Os‐C(W_5_) (Figure 3E,F) resulted in cells with a rounder morphology, visible budding scars, membrane tears and invaginations, as well as the presence of scattered debris.

**FIGURE 4:**
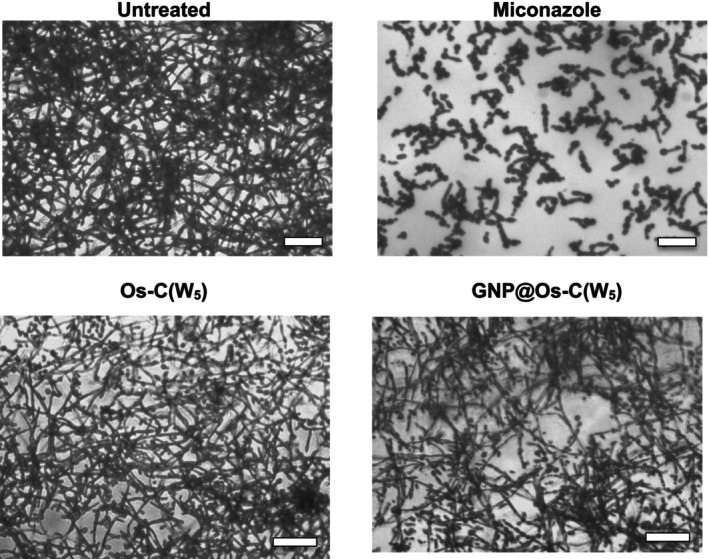
Micrographs of biofilm inhibiting activity of miconazole, Os‐C(W_5_) and GNP@Os‐C(W_5_). Morphology of *C. albicans* biofilms stained with CV following exposure of *C. albicans* cells to ddH_2_O, 125 μM miconazole, 75 μM of Os‐C(W5) and GNP@Os‐C(W_5_) for 24 h. Scale bar = 20 μm.

The inhibitory effects of GNP@Os‐C(W_5_) on *C. albicans* biofilm formation were further evaluated microscopically after CV staining. Untreated *C. albicans* cells established a robust, dense, and highly interconnected hyphal network characterized by extensive filamentous growth (Figure 4). In contrast, miconazole treatment displayed predominantly isolated and clustered microcolonies with minimal visible hyphal development. Samples treated with Os‐C(W5) and GNP@Os‐C(W5) showed visibly less dense biofilm architecture and fewer apparent hyphal structures relative to the untreated control. Overall, biofilm density was reduced and hyphal development appears to be suppressed.

### 3.5 Os‐C(W_5_) and GNP@Os‐C(W_5_) Are Not Cytotoxic Toward HaCaT Cells

**FIGURE 5:**
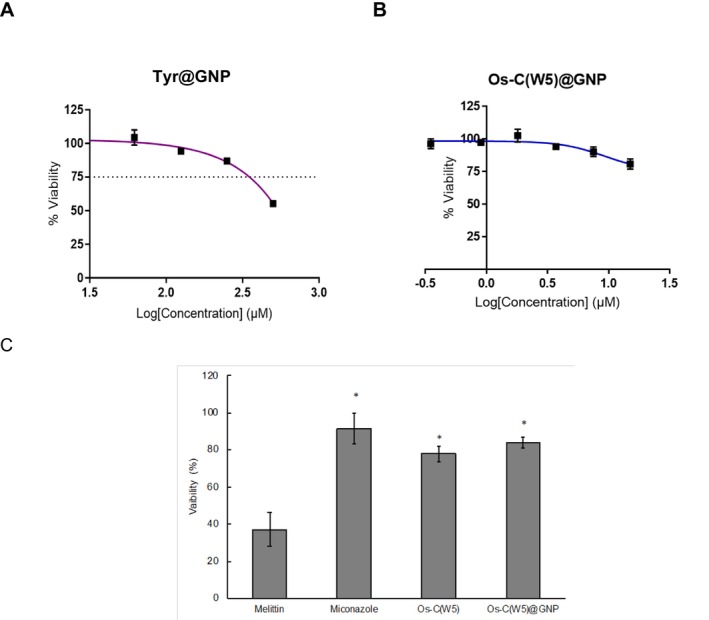
Cytotoxicity of (A) 31–500 μM Tyr@GNP, (B) 0.35–15 μM Os‐C(W5)@GNP and C) 15 μM melittin, miconazole, Os‐C(W5) and GNP@Os‐C(W5) against HaCat cells. After 24 h of exposure, cell viability was determined with the MTT assay. Data is the mean of three independent experiments in triplicate ± SEM For (C) compared with melittin, statistical significance indicated by * representing *p* < 0.01.

The cytotoxicity of 31–500 μM Tyr@GNP and 0.35–15 μM GNP@Os‐C(W_5_) was reduced to 55% and 84% for Os‐C(W_5_) and GNP@Os‐C(W_5_) at 500 μM and 15 μM respectively (Figure 5A,B). At 15 μM the cytotoxicity of GNP@Os‐C(W_5_) was compared with miconazole, melittin (a cytolytic peptide), and Os‐C(W_5_). As expected, melittin reduced HaCaT cell viability to 37.27% ± 9.5% (Figure 5c). In contrast, miconazole, Os‐C(W_5_), and GNP@Os‐C(W_5_) showed limited cytotoxicity, with no significant differences between miconazole, Os‐C(W_5_), and GNP@Os‐C(W_5_).

### 3.6 Conjugation Onto GNPs Protects Os‐C(W_5_) From Proteolytic Degradation

**FIGURE 6:**
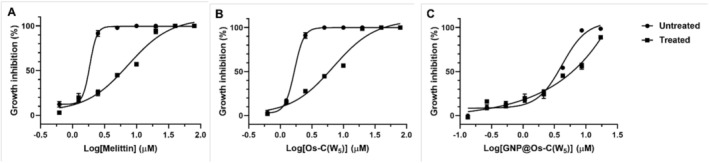
Stability of melittin, Os‐C(W_5_) and GNP@Os‐C(W_5_) following pre‐treatment with 1.86 U trypsin for 1 h prior to the determination of antiplanktonic activity against *C. albicans* . Percentage inhibition is shown for cells exposed to a final concentration of 0.25 μM–32 μM melittin, 0.125 μM–16 μM Os‐C(W_5_) and 0.054 μM–16 μM GNP@Os‐C(W_5_). Data represents the mean of three independent experiments ± SD.

The susceptibility of AMPs to protease degradation limits clinical application and in several studies, trypsin, a serine protease, was used to evaluate the stability of AMPs [16, 38]. The effect of GNP conjugation on the stability of Os‐C(W_5_) was determined in the presence of trypsin (Figure 6). Both melittin and Os‐C(W_5_) possess several trypsin proteolytic sites; therefore, higher peptide concentrations were required to inhibit the growth of planktonic *C. albicans* . In contrast, conjugating Os‐C(W_5_) to GNPs markedly improved its resistance to trypsin‐mediated degradation, indicating enhanced proteolytic stability.

### 3.7 Antioxidant Activity Is Lost Upon Conjugation to GNPs

**FIGURE 7:**
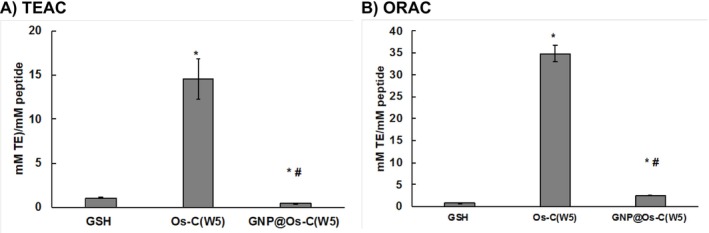
Antioxidant activity of GSH, Os‐C(W_5_) or GNP@Os‐C(W_5_) using the (A) TEAC and (B) ORAC assay. Antioxidant activity was calculated for GSH, Os‐C(W_5_) or GNP@Os‐C(W_5_) as Trolox Equivalent (TE) ratios mM TE/mM peptide for GSH, Os‐C(W_5_) or GNP@Os‐C(W_5_). Data represents the mean of three independent experiments ± SEM Statistical difference *compared with GSH and ^#^between Os‐C(W_5_) and GNP@Os‐C(W_5_), (*p* < 0.05).

Os and Os‐C have previously been identified as multifunctional peptides [32, 39]. To observe whether conjugation affects the antioxidant activity of Os‐C(W_5_), TEAC and ORAC assays were conducted. For the TEAC assay (Figure 7A), Os‐C(W_5_) exhibited markedly higher activity than GSH, showing a 15‐fold increase in relative antioxidant capacity (mM TE/mM peptide), a statistically significant difference. In contrast, GNP@Os‐C(W_5_) displayed substantially reduced activity (0.36 ± 0.09 mM TE/mM peptide), falling below that of GSH and indicating a loss of antioxidant function following GNP conjugation. For both assays, GNP@Tyr displayed no detectable antioxidant function (data not shown), confirming that the effects observed were peptide dependent. Consistent with the TEAC assay, ORAC measurements (Figure 7B) demonstrated that Os‐C(W_5_) exhibited significantly greater antioxidant activity than GSH. Conjugation to GNPs resulted in a 14.36‐fold reduction, yet GNP@Os‐C(W_5_) still retained antioxidant capacity, exhibiting significantly higher ORAC than GSH, though much lower than free Os‐C(W_5_).

### 3.8 Os‐C and Its Derivatives Have Reduced Nitric Oxide Scavenging Activity

**FIGURE 8:**
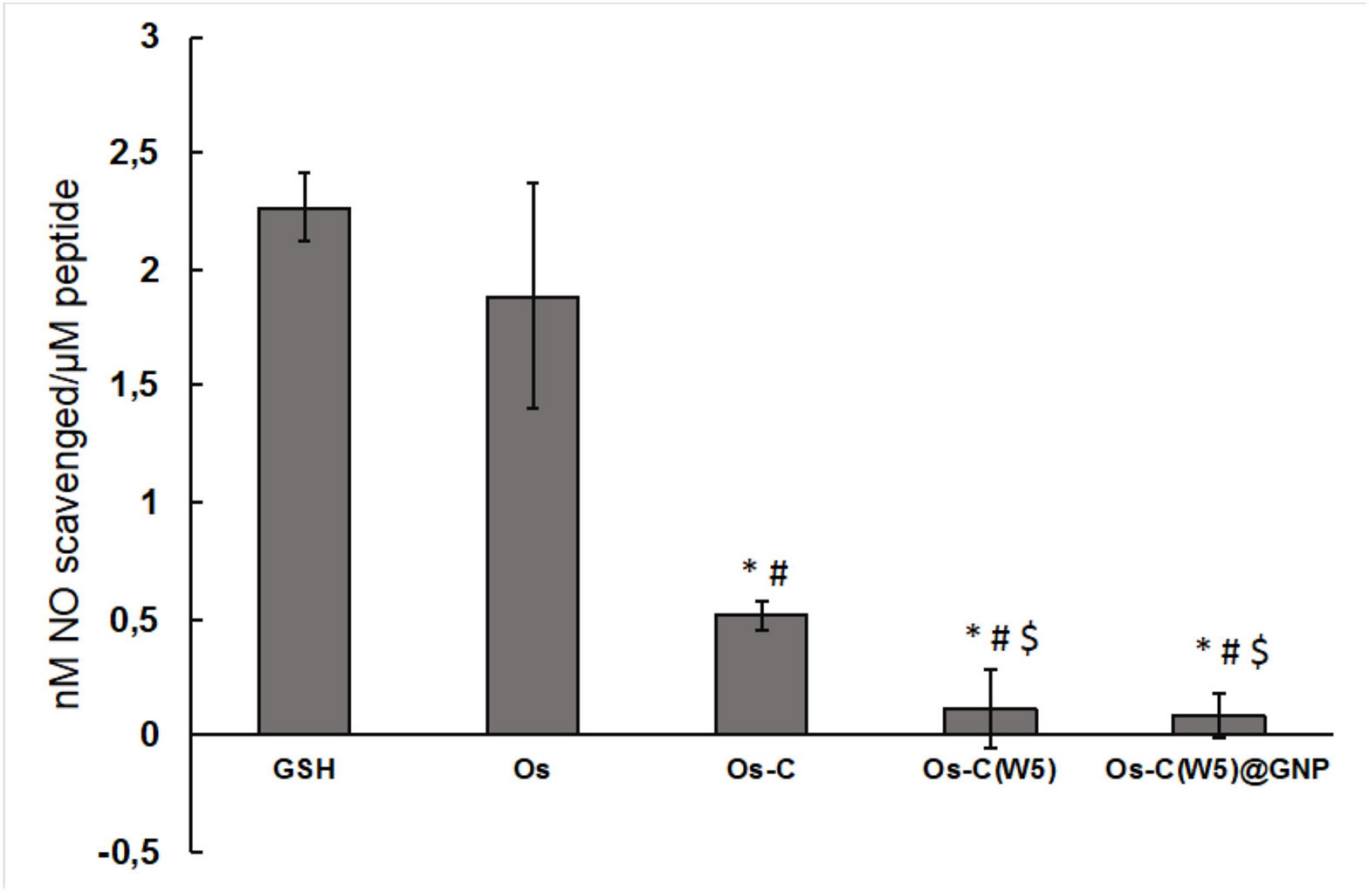
Scavenging of SNP generated NO by GSH, Os, Os‐C, Os‐C(W_5_) and GNP@Os‐C(W_5_). The nM NO scavenging ability of GSH, Os, Os‐C, Os‐C(W_5_) and GNP@Os‐C(W_5_) per μM peptide. Data represents the mean of three independent experiments in triplicate ± SEM Statistical difference * GSH compared with all peptides, ^#^ Os compared with Os‐C, Os‐C(W_5_) and GNP@Os‐C(W_5_) and ^$^ Os‐C compared with Os‐C(W_5_) and GNP@Os‐C(W_5_), (*p* < 0.05).

To evaluate the direct NO scavenging capabilities of AMPs, SNP was quantified with the Griess reaction (Figure 8). Both GSH and Os effectively scavenged NO. As previously reported, the NO scavenging activity of Os‐C is reduced compared with Os [39]. Relative to Os‐C, both Os‐C(W_5_) and GNP@Os‐C(W_5_) exhibit further reduced NO scavenging activity, with no significant difference observed between Os‐C(W_5_) and GNP@Os‐C(W_5_).

## 4 Discussion

We previously demonstrated that C‐terminal tryptophan end‐tagging of Os‐C resulted in Os‐C(W_5_), an analog that had increased antifungal activity in physiologically relevant salt conditions [7]. Nonetheless, it was necessary to address the susceptibility of Os‐C(W_5_) to proteases. To address this limitation, a peptide‐mediated GNP synthesis approach was adapted as described by Ozaki et al. (2020) to synthesize GNP@Os‐C(W_5_), with GNP@Tyr included as a nanoparticle control [23]. Previous studies have shown that N‐terminal tryptophan residues can drive Au^3+^ reduction and GNP formation without secondary reagents, with nanoparticle size increasing as a function of increasing tryptophan residues [19, 23]. Importantly, peptides containing multiple tryptophan residues generated nanoparticles larger than 10 nm in size, a range associated with reduced mammalian cytotoxicity [40, 41, 42]. Furthermore, this approach offers practical advantages, as it avoids toxic reagents while enabling simple, rapid synthesis with high peptide‐nanoparticle binding efficiency.

Tyrosine‐mediated synthesis produced a high yield of control GNPs of uniform size, consistent with previous reports that deprotonated tyrosine efficiently reduces Au^3+^ [12] whereas the phenolic group of tyrosine facilitates favorable interactions with the gold surface generating GNP@Tyr. The synthesized GNP@Tyr had an average diameter of 16.2 ± 2.0 nm, similar to the 15 nm GNP@Tyr obtained by Jia, et al. (2019) [12]. This nanoparticle control displayed no antifungal, antioxidant, or NO scavenging activity, and consequently this scaffold does not contribute intrinsically to the observed bioactivity. Therefore, it can be inferred that the biological activity of GNP@Os‐C(W_5_) is predominantly peptide‐driven, whereas the nanoparticle scaffold acts primarily as an inert carrier that may enhance peptide presentation and local concentration rather than contributing intrinsic antifungal activity.

Compared with GNP@Tyr, GNP@Os‐C(W_5_) particles were slightly smaller (14.22 ± 0.31 nm), suggesting that amino acid composition, tryptophan positioning and the overall peptide sequence influence nanoparticle nucleation and growth [43]. In particular, aromatic residues such as tryptophan and tyrosine have been implicated in Au^3+^ reduction and stabilization [12, 19], whereas the broader peptide sequence likely contributes to nanoparticle growth and surface passivation, with positively charged residues such as lysine and arginine potentially participating in electrostatic interactions with the gold surface, whereas glycine may primarily confer conformational flexibility rather than directly contributing to binding [44]. Fourier‐transform infrared spectroscopy indicated possible interactions involving lysine side chains and the nanoparticle surface, suggesting a potential role for these residues in adsorption and early‐stage nucleation events [45]. The precise binding modes and spatial orientation of the peptide on the nanoparticle surface are unknown and will be elucidated in future studies using *in silico* molecular dynamics [23]. Furthermore, in biologically relevant environments such as wounds, peptide stability and the influence of salt concentration, ionic strength, pH, and temperature on its release kinetics, antifungal efficacy, and interactions with the *C. albicans* cell wall should be evaluated.

The secondary structure of Os‐C(W_5_) was assessed by CD in aqueous (Tris) and membrane‐mimetic (SDS) environments [46, 47]. Os‐C(W_5_) adopts a predominantly disordered conformation, although it exhibits approximately 13.5% higher β‐strand content relative to Os‐C, which may be attributed to tryptophan clustering effects that promote transient local ordering through aromatic stacking and hydrophobic collapse [7]. This observation is consistent with reports on other AMPs where disordered or partially structured AMPs have been shown to facilitate nanoparticle formation and simultaneously preserve membrane activity, supporting a dual functional role in bio‐nano systems [44, 48].

Upon conjugation to GNPs, the overall CD signal of Os‐C(W_5_) was significantly attenuated and spectrally flattened, without the emergence of canonical α‐helical or β‐sheet minima. This reduction in ellipticity may be attributed to adsorption‐induced restriction of conformational flexibility and reduced backbone mobility, together with nanoparticle‐associated optical artifacts, rather than a true structural rearrangement [49, 50]. These effects can reduce the magnitude of the CD signal while preserving the underlying disordered conformational ensemble. Consistent with this, AMPs frequently retain activity in disordered states, where membrane interaction depends more on amphipathicity and surface presentation than on stable secondary structure [51]. In addition, interaction involving aromatic side chains, particularly indole groups of terminal tryptophan residues, may contribute to local electronic perturbations, including quenching or redistribution of electronic transitions, thereby further diminishing observable ellipticity [52]. Despite this attenuation, the absence of newly emergent α‐helical or β‐sheet signatures indicates that GNP binding does not drive a global disorder‐to‐order transition in Os‐C(W_5_), and the peptide remains largely disordered, similar to the parent Os‐C sequence [7]. Notably, although limited literature exists on non‐covalent AMP‐GNP conjugates specifically, studies on covalently attached peptide‐nanoparticle systems report CD spectra that closely resemble those of the free peptide [53], indicating that immobilization alone does not necessarily induce changes in secondary structure.

Both Os‐C(W_5_) and GNP@Os‐C(W_5_) exhibited antifungal activity and induced morphological changes in *C. albicans* . Although conjugation of Os‐C(W_5_) onto GNPs resulted in a ten‐fold reduction in antiplanktonic inhibition relative to free Os‐C(W_5_), antibiofilm efficacy was largely retained. The relationship between the MIC and MFC values suggested differences in antifungal activity among the treatments. A higher MFC/MIC ratio for Os‐C(W_5_) (4.1) suggested mainly fungistatic activity, whereas the lower ratios observed for GNP@Os‐C(W_5_) (1.4) and miconazole (1.6) were consistent with fungicidal activity [54]. SEM analysis at MIC_50_ values revealed greater ultrastructural damage following treatment with GNP@Os‐C(W_5_) compared with Os‐C(W_5_), including significant membrane irregularities and surface indentations. Following exposure of *C. albicans* to Os‐C(W_5_), similar surface alterations associated with membrane stress and ROS‐mediated cellular damage were observed [7]. Although ROS production and membrane permeabilization were not directly quantified in the present study, these observations suggest that membrane‐associated damage is attributed to this mechanism of action of the released peptide, Os‐C(W_5_). Furthermore, consistent with the CD findings, differences in planktonic antifungal activity are unlikely to arise from a major conformational change in the peptide following nanoparticle conjugation. The absence of a disorder‐to‐order transition indicates that the altered fungicidal behavior of GNP@Os‐C(W_5_) is more plausibly attributable to nanoparticle‐mediated effects including altered peptide presentation [44] and/or time dependent release [55].

Despite reduced planktonic potency, antibiofilm efficacy was retained following conjugation. Although BIC_50_ values for biofilm cell viability showed only a slight, statistically non‐significant reduction, the BIC_50_ for biofilm biomass was reduced, indicating a differential effect on biofilm‐associated endpoints rather than a uniform response across viability and biomass measurements. Antiplanktonic effects were evident within 3 h, whereas maximal biofilm inhibition occurred after 24 h, suggesting the temporal difference may indicate that conjugation to GNPs alters the presentation, release, or interaction dynamics of Os‐C(W_5_) to the different growth phases of *C. albicans* . In this study, the underlying mechanisms were not directly investigated. In addition, antifungal activity was limited to one strain and broader validation against a diverse panel of clinical isolates, including antifungal‐resistant strains, is required to confirm and strengthen relevance.

Biofilm inhibition remained comparable to values previously reported for Os‐C(W_5_), indicating that conjugation to GNPs did not diminish the antibiofilm activity of the peptide [7, 56]. Microscopic analysis qualitatively indicated that exposure to 75 μM GNP@Os‐C(W_5_) was associated with altered *C. albicans* biofilm architecture, including reduced apparent biofilm density and fewer visible hyphal structures compared with the untreated control. However, these observations are qualitative and were not subjected to image‐based quantification but are consistent with disruption of early biofilm development processes, particularly cell adhesion and hyphal transition, which are critical steps in mature biofilm formation [57]. Similar effects on hyphal development have been reported for other AMPs, including LL‐37 and psoriasin, which limit early cell adhesion, a critical step in biofilm establishment [58, 59, 60]. As release kinetics were not investigated in the present study, further studies are required to clarify the contribution of peptide availability and delivery dynamics to the observed biofilm effects.

For therapeutic application, AMPs must effectively eradicate infections while minimizing mammalian cytotoxic effects. Unlike melittin, an AMP that induces erythrocyte hemolysis [61], Os‐C(W_5_) is non‐hemolytic [7]. The cytotoxicity of Tyr@GNP, Os‐C(W_5_), and GNP@Os‐C(W_5_) was determined using the HaCaT keratinocyte cell line, a well‐established in vitro model for evaluating the safety of peptides developed for wound healing applications. Cytotoxcity was minimal and although an IC_50_ could not be calculated, it can be concluded that GNP@Os‐C(W_5_) is selective for *C. albicans* .

The primary objective of conjugating Os‐C(W_5_) to GNPs was to enhance resistance to proteases. It was seen that conjugation onto the GNP surface reduced peptide degradation, likely due to steric hindrance and limited protease access to cleavage sites. This is in accordance with previous literature [16, 44], where several peptides on the GNP surface formed a protective peptide layer that limited protease accessibility.

The dual antimicrobial‐antioxidant functionality of Os‐C(W_5_) and GNP@Os‐C(W_5_) was evaluated, as antioxidant activity is strongly associated with tryptophan and tyrosine residues, as shown in studies where short peptides, rich in these amino acids, exhibited strong radical scavenging [62, 63]. Accordingly, Os‐C(W_5_), containing five tryptophan and three tyrosine residues, exhibited high antioxidant activity at 1 μM (TEAC: 14.59 μM TE; ORAC: 34.83 μM TE). Conjugation to GNPs significantly reduced this activity, likely due to masking of redox‐active residues and possible involvement of tryptophan in peptide binding through non‐covalent hydrogen bonding [64].

Nitric oxide scavenging is largely dependent on cysteine thiols, which form S‐nitrosylated derivatives that play a central role in regulating nitrosative stress [65, 66]. Accordingly, cysteine‐containing peptides (GSH and Os) exhibited strong NO scavenging activity, whereas Os‐C and Os‐C(W_5_), which lack cysteine, displayed reduced or negligible NO scavenging effects. The limited activity in Os‐C is consistent with the weak NO‐scavenging activity of branched‐chain amino acids such as isoleucine, leucine, and valine [67], explaining the minimal NO scavenging by Os‐C(W_5_) and GNP@Os‐C(W_5_).

## 5 Conclusion

In conclusion, Os‐C(W_5_) was effectively associated with GNPs, with a seemingly high conjugation efficiency. Compared with Os‐C(W_5_), GNP@Os‐C(W_5_) exhibited reduced planktonic activity and maintained antibiofilm inhibition, with a reduction in biofilm biomass. The conjugate was non‐cytotoxic to mammalian HaCat cells. Planktonic antifungal activity was associated with morphological changes in *C. albicans* , including membrane irregularities and surface damage, whereas biofilm assays indicated changes in biofilm density and hyphal formation. Inhibition of biofilm formation resulted in less dense biofilms and reduced hyphal formation. Compared with free Os‐C(W_5_), GNP@Os‐C(W_5_) was more resistant to trypsin proteolysis. GNP conjugation resulted in the loss of Os‐C(W_5_) antioxidant activity, whereas the NO scavenging activity of Os‐C was lost after tagging and nanoparticle formation. Notably, the phenolic groups of tyrosine‐capped GNPs provide stable, non‐covalent anchoring, suggesting GNP@Tyr could serve as an inert platform for conjugating and immobilizing other AMPs for the further development of peptide‐based antimicrobial nanomaterials.

## Author Contributions

**H. Taute and M. J. Bester:** conceived and designed the study; **P. J. Palm, R. R. Chirombo, and C. K. Chiramba:** performed the experiments and analyzed the data; **J. C. Serem, M. van der Walt, and A. R. M. Gaspar:** contributed to manuscript preparation and revision. All authors reviewed and approved the final manuscript.

## Funding

This research was funded by the National Research Foundation, Thuthuka Fund (grant no: 129781).

## Conflicts of Interest

The authors declare no conflicts of interest.

## Supporting information


**Figure S1:** HPLC analysis of Os‐C.
**Figure S2:** Mass spectrometry analysis of Os‐C.
**Figure S3:** HPLC analysis of Os‐C(W_5_).
**Figure S4:** Mass spectrometry analysis of Os‐C(W_5_).
**Figure S5:** Effect of N‐terminal Trp tagging of Os‐C on the formation, stability, and average diameter of the formed GNPs. A concentration of 150 μM (W5)Os‐C was added to 1 mM HAuCl_4_.3H_2_O and mixed for 24 h, then the (A) UV–Vis spectra were determined directly after synthesis and 24 h later. The (B) morphology of the generated GNPs was evaluated with transmission electron microscopy. The scale bar represents 100 nm. The C) UV–Vis spectra of Os‐C(W5) showed to remain stable after 7 days.
**Figure S6:** The effect of Os‐C(W5) concentration on Os‐C(W5)@GNP synthesis. (A) A representative UV–Vis spectrum of 1 mM L‐Tyr, along with concentrations of 38 μM, 76 μM, and 150 μM of Os‐C(W5), utilized to generate the corresponding GNPs.
**Figure S7:** Inhibition of *C. albicans* biofilm formation by miconazole and Os‐C(W5)@GNP. The cell viability was determined with the resazurin assay and cell biomass was determined with the crystal violet assay. Data is the mean of three independent experiments done in triplicate ± SEM.
**Table S1:** Fungicidal activity of Os‐C(W5) and GNP@Os‐C(W5) in planktonic *C. albicans.*

## Data Availability

The data that support the findings of this study are available from the corresponding author upon reasonable request.
