# Supplementary material for: Gold Nanoparticles Coated With the Antimicrobial Peptide Os‐C(W5): Anticandidal and Biological Activity
Source: J Pept Sci. 2026 Aug 2;32(9):e70117. doi: 10.1002/psc.70117 (PMC13430083; doi:10.1002/psc.70117)

**Gold nanoparticles coated with the antimicrobial peptide Os-C(W5): Anticandidal and biological activity**

P. J. Palm^a*^, R.R. Chirombo^a*^, C.K. Chiramba^b^, J.C. Serem ^a^, H. Taute ^a^, A.R.M. Gaspar^b^ and M.J. Bester ^a^ *

^a^ Department of Anatomy, Faculty of Health Sciences, University of Pretoria, 0002, South Africa

^b^ Department of Biochemistry, Genetics and Microbiology, Faculty of Natural and Agricultural Sciences, University of Pretoria, 0002, South Africa

^*^Both contributed equally to this manuscript

**Corresponding author**: Department of Anatomy, Faculty of Health Sciences, University of Pretoria, 0002, South Africa. megan.bester@up.ac.za


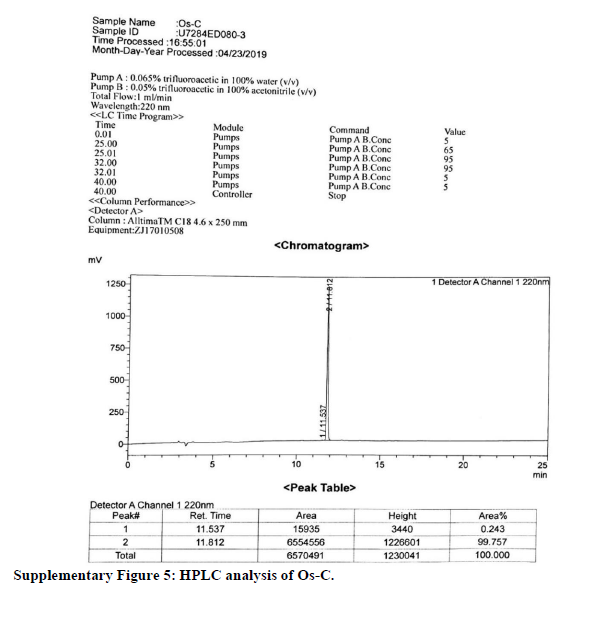
Supplementary Figure 1: HPLC analysis of Os-C


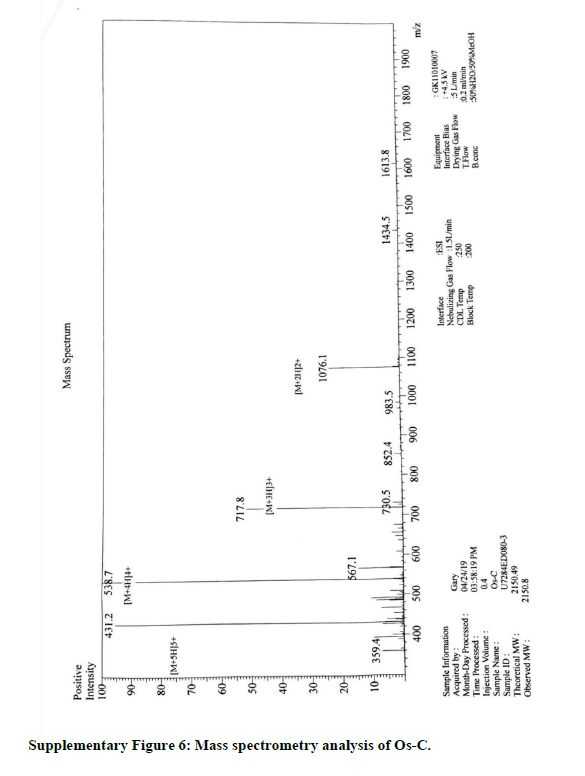
Supplementary Figure 2: Mass spectrometry analysis of Os-C


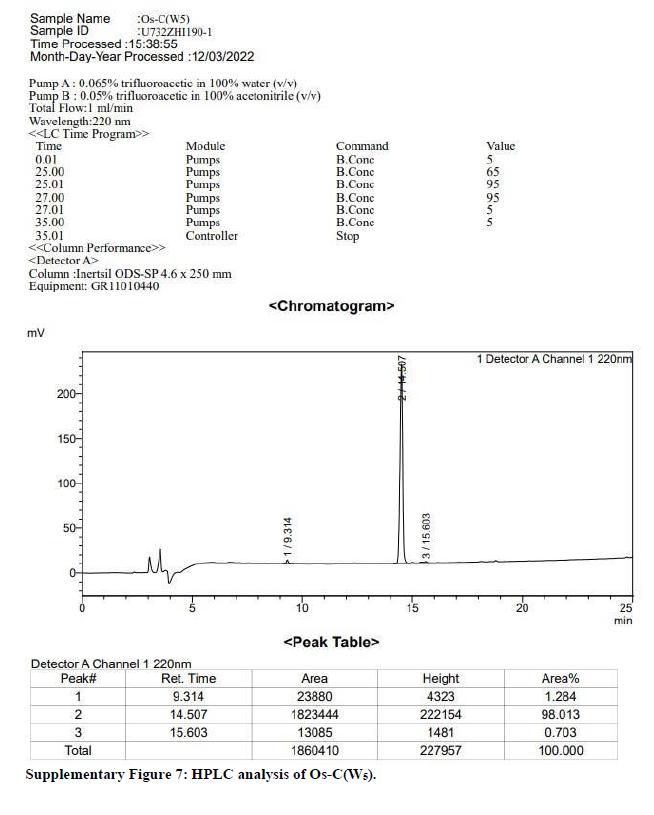
Supplementary Figure 3: HPLC analysis of Os-C(W_5_)


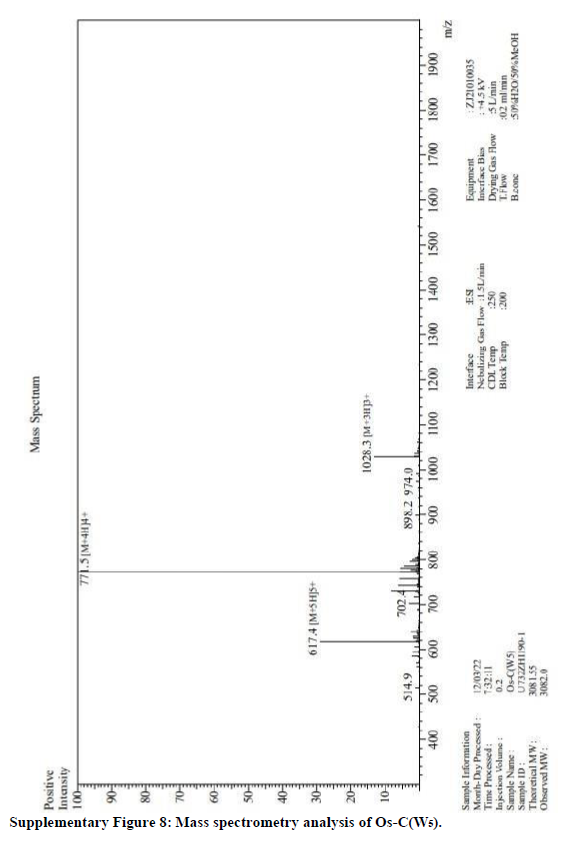
 Supplementary Figure 4: Mass spectrometry analysis of Os-C(W_5_)

| **GNP@(W5)Os-C** | | |
| --- | --- | --- |
| **A** | **** | |
| **B** | | **C** |
| **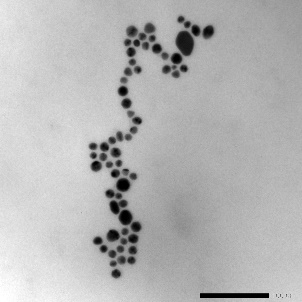**  **100 nm** | |  |
| Supplementary Figure 5: Effect of N-terminal Trp tagging of Os-C on the formation, stability, and average diameter of the formed GNPs. A concentration of 150 µM (W5)Os-C was added to 1 mM HAuCl_4_^.^3H_2_O and mixed for 24 hours, then the A) UV-Vis spectra were determined directly after synthesis and 24 hours later. The B) morphology of the generated GNPs was evaluated with transmission electron microscopy. The scale bar represents 100 nm. The C) UV-Vis spectra of Os-C(W5) showed to remain stable after 7 days. | | |

**
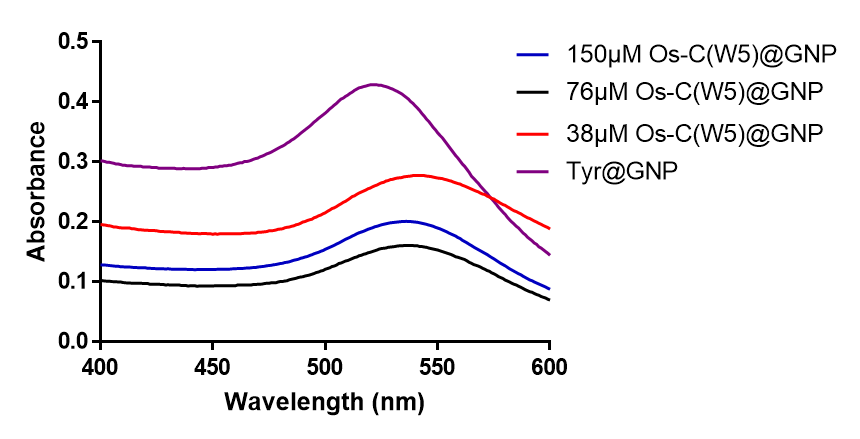
Supplementary Figure 6**: The effect of Os-C(W5) concentration on Os-C(W5)@GNP synthesis. A) A representative UV-Vis spectrum of 1 mM L-Tyr, along with concentrations of 38 µM, 76 µM, and 150 µM of Os-C(W5), utilised to generate the corresponding GNPs

| **BIOFILM INHIBITION** | |
| --- | --- |
| **Miconazole** | |
| **Cell viability** | **Biomass** |
| **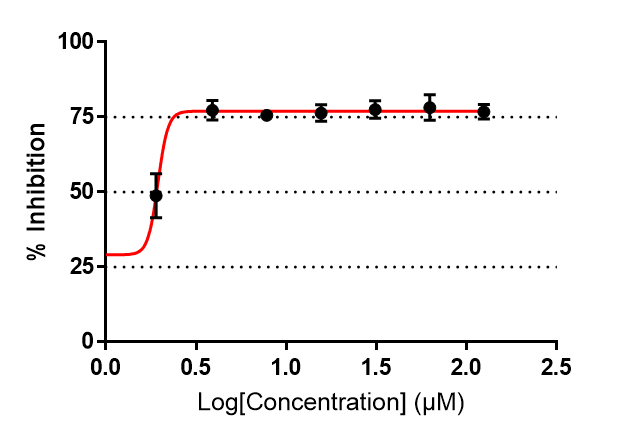** | **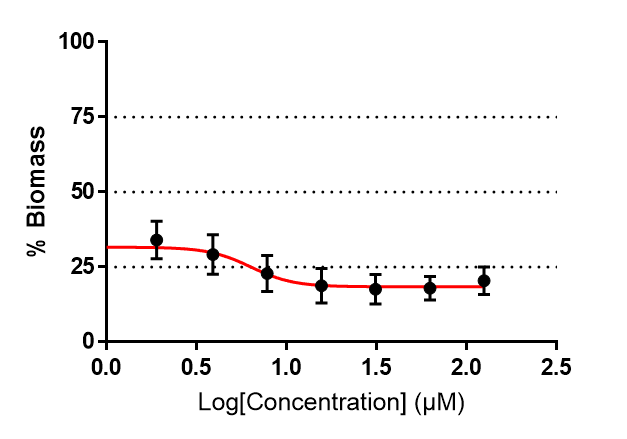** |
| **Os-C(W5)@GNP** | |
| **Cell viability** | **Biomass** |
| **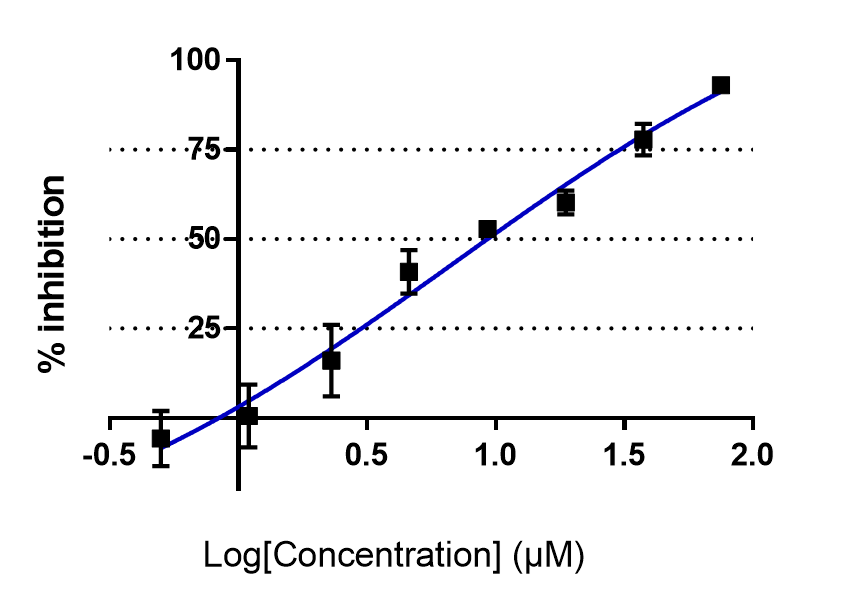** | **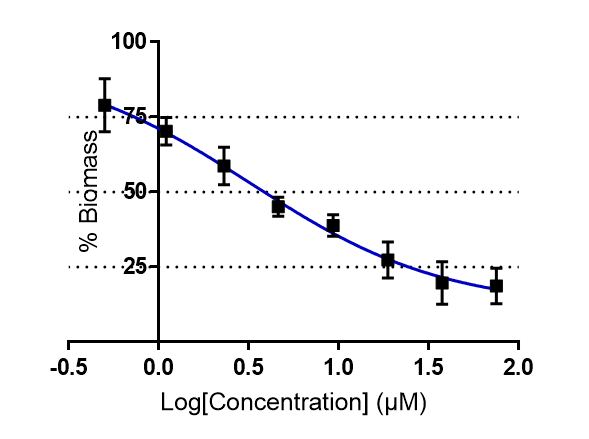** |

**Supplementary Figure 7: Inhibition of C. albicans biofilm formation by miconazole and Os-C(W5)@GNP**. The cell viability was determined with the resazurin assay and cell biomass was determined with the crystal violet assay. Data is the mean of three independent experiments done in triplicate ± SEM

**Supplementary Table 1:** Fungicidal activity of Os-C(W5) and GNP@Os-C(W5) in planktonic C. albicans


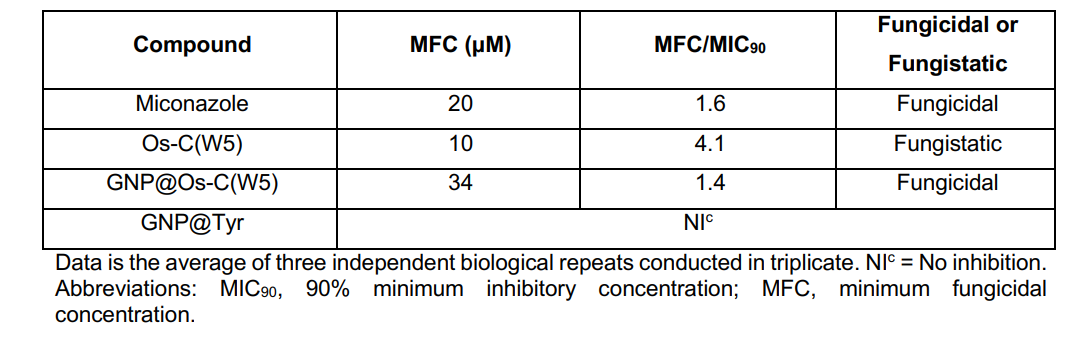

Supplement: Supplementary file 1 — Figure S1: HPLC analysis of Os‐C. Figure S2: Mass spectrometry analysis of Os‐C. Figure S3: HPLC analysis of Os‐C(W5). Figure S4: Mass spectrometry analysis of Os‐C(W5). Figure S5: Effect of N‐terminal Trp tagging of Os‐C on the formation, stability, and average diameter of the formed GNPs. A concentration of 150 μM (W5)Os‐C was added to 1 mM HAuCl4.3H2O and mixed for 24 h, then the (A) UV–Vis spectra were determined directly after synthesis and 24 h later. The (B) morphology of the generated GNPs was evaluated with transmission electron microscopy. The scale bar represents 100 nm. The C) UV–Vis spectra of Os‐C(W5) showed to remain stable after 7 days. Figure S6: The effect of Os‐C(W5) concentration on Os‐C(W5)@GNP synthesis. (A) A representative UV–Vis spectrum of 1 mM L‐Tyr, along with concentrations of 38 μM, 76 μM, and 150 μM of Os‐C(W5), utilized to generate the corresponding GNPs. Figure S7: Inhibition of C. albicans biofilm formation by miconazole and Os‐C(W5)@GNP. The cell viability was determined with the resazurin assay and cell biomass was determined with the crystal violet assay. Data is the mean of three independent experiments done in triplicate ± SEM. Table S1: Fungicidal activity of Os‐C(W5) and GNP@Os‐C(W5) in planktonic C. albicans. [file PSC-32-e70117-s001.docx]
